# Evolutionary Inverse Material Identification: Bespoke Characterization of Soft Materials Using a Metaheuristic Algorithm

**DOI:** 10.3389/frobt.2021.790571

**Published:** 2022-01-14

**Authors:** Michele Di Lecce, Onaizah Onaizah, Peter Lloyd, James H. Chandler, Pietro Valdastri

**Affiliations:** Science and Technology of Robots in Medicine (STORM) Laboratory, School of Electronics and Electrical Engineering, University of Leeds, Leeds, United Kingdom

**Keywords:** soft robots material and design, magnetic actuation, hyperelastic models, material characterization and modeling, evolutionary algorithm, inverse optimization, CMA-ES optimization

## Abstract

The growing interest in soft robotics has resulted in an increased demand for accurate and reliable material modelling. As soft robots experience high deformations, highly nonlinear behavior is possible. Several analytical models that are able to capture this nonlinear behavior have been proposed, however, accurately calibrating them for specific materials and applications can be challenging. Multiple experimental testbeds may be required for material characterization which can be expensive and cumbersome. In this work, we propose an alternative framework for parameter fitting established hyperelastic material models, with the aim of improving their utility in the modelling of soft continuum robots. We define a minimization problem to reduce fitting errors between a soft continuum robot deformed experimentally and its equivalent finite element simulation. The soft material is characterized using four commonly employed hyperelastic material models (Neo Hookean; Mooney–Rivlin; Yeoh; and Ogden). To meet the complexity of the defined problem, we use an evolutionary algorithm to navigate the search space and determine optimal parameters for a selected material model and a specific actuation method, naming this approach as Evolutionary Inverse Material Identification (EIMI). We test the proposed approach with a magnetically actuated soft robot by characterizing two polymers often employed in the field: Dragon Skin™ 10 MEDIUM and Ecoflex™ 00-50. To determine the goodness of the FEM simulation for a specific set of model parameters, we define a function that measures the distance between the mesh of the FEM simulation and the experimental data. Our characterization framework showed an improvement greater than 6% compared to conventional model fitting approaches at different strain ranges based on the benchmark defined. Furthermore, the low variability across the different models obtained using our approach demonstrates reduced dependence on model and strain-range selection, making it well suited to application-specific soft robot modelling.

## Introduction

Over the last few decades, there has been growing interest in the field of soft robotics (Lipson, 2014; Rus and Tolley, 2015). These robots offer many advantages over their rigid body counterparts, with the ability to traverse complex trajectories to reach previously inaccessible areas, deform both actively and passively in multiple directions, and interact safely within delicate environments (e.g., with biological tissues). Furthermore, they often represent simpler fabrication and assembly with respect to rigid robots with joints; being molded in monolithic material designs (Chandler et al., 2020), with embedded strain limiting materials (Mosadegh et al., 2014; Polygerinos et al., 2015) or with the addition of functional components (e.g., magnetic particles) (Kim et al., 2019; Lloyd et al., 2019). These advantages are afforded due to the highly compliant nature of the materials from which they are typically made, and have made soft robots (SRs) a popular choice for small-scale medical and surgical instrumentation (Cianchetti et al., 2014, 2018; da Veiga et al., 2020); from common grasping tasks (Zhang et al., 2017b), endoscopic (Chauhan et al., 2021; Liu et al., 2021) and minimally invasive surgery (Edelmann et al., 2017; Oliver-Butler et al., 2017; Jeon et al., 2019) to microfluidic platforms in order to stimulate and sort cells (Zhang et al., 2017c; Onaizah et al., 2020).

Actuation of SRs is possible using numerous methods, including, for example, pneumatic, hydraulic, mechanical, chemical and magnetic approaches (Burgner-Kahrs et al., 2015; Le et al., 2016; Thuruthel et al., 2018). However, in the case of medical and surgical applications, the use of magnetic actuation is particularly advantageous as it allows for improved device scalability since mechanical transmission and on-board power and electronics can be removed (Abbott et al., 2020; da Veiga et al., 2020). Forces and torques are generated wirelessly due to ferromagnetic bodies embedded inside the robot interacting with a local or global magnetic field often generated by electromagnetic coils (Kummer et al., 2010; Petruska and Abbott, 2014; Sikorski et al., 2017) or permanent magnets (Pittiglio et al., 2020). Magnetically actuated soft robots (MASRs) can be produced in two different ways: a soft elastomeric matrix can be created using magnetic powder mixed with the soft materials before the robot is molded (Lum et al., 2016; Zhang C. et al., 2017; Kim et al., 2019; Lloyd et al., 2019) or a permanent magnet can be placed inside a cavity of the already fabricated SR (Jeon et al., 2019; Tariverdi et al., 2021). Each approach has its advantages and disadvantages with permanent magnets offering a higher magnetic material density and thus larger forces and torques for a given external field but also adding a rigid domain to the robot. Using an elastomeric matrix, on the other hand, offers the advantages of maintaining an entirely compliant device, however, introduces changes to the base elastomer material properties and thus characterization is limited to that specific matrix (da Veiga et al., 2021).

As with SRs actuated using alternative means, MASRs are manufactured using soft elastomeric polymers with elastic moduli values close to those of biological tissues. While these highly compliant materials support safer tissue interactions in surgical applications, they are much harder to model and thus predict their behavior. Under actuation, the hyperelastic material can produce highly nonlinear deformations that cannot be resolved using techniques conventionally used in robotics. As noted in the research conducted by Chen and Wang (2020), the preferable way to test and optimize a SR during the early stages of the design is through finite element analysis (FEA). If a FEA model is well defined, it can accelerate the process of design optimization, reducing the need for repeated experimentation and thus lowering the cost.

Several material models of varying complexity have been proposed (Mihai and Goriely, 2017), including: Neo Hookean, Yeoh, Mooney-Rivlin and Ogden models. These models aim to deliver a relationship between the principal stresses and the deformation of the material when subject to a mechanical load. The most popular procedure for characterizing a specific hyperelastic material, under the hypothesis of isotropy and incompressibility, involves a mechanical tensile test up to material failure to collect the stress-strain data (Marechal et al., 2020). While tensile testing based experiments are the most popular way to characterize the material model, these have some limitations including that the strain produced in the gauge area of the specimen may be larger than the strain measured globally (Krautz et al., 2017). One way to solve this problem is through image correlation by observing the strain locally in the gauge area (Hartmann et al., 2003; Meunier et al., 2008). While this technique seems promising, it may not be widely accessible. Furthermore, tensile tests miss information like shear stress that often requires the use of more complex testing, such as equiaxial testing (Steinmann et al., 2012) or volumetric testing (Horgan and Murphy, 2009) which requires multiple experimental platforms and can be expensive and time-consuming.

**FIGURE 1:**
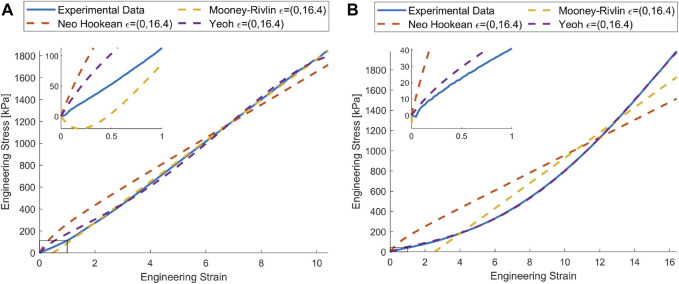
When the material models are trained using the entire set of tensile data, the error from the model in the region of interest may be considerable. In this case, we highlight this effect by zooming into a smaller strain range (100%), for **(A)** Dragon Skin™ 10 MEDIUM and **(B)** Ecoflex™ 00-50 where there is a significant variance between the models and experimental data. Ogden model excluded for readability [Data and the Code provided by Marechal et al. (2020)].

In addition, soft materials will deform several times their original length before the fracture point is reached during a mechanical test. While this data is relevant for some applications such as pneumatically actuated SRs (Mosadegh et al., 2014), in other applications, the forces and torques applied on the material generates only a fraction of the deformation experienced by the specimens during the tensile test (Steck et al., 2019). Furthermore, using the entire tensile test dataset to fit the parameters for lower order models can introduce considerable errors in the region of interest as the models may overestimate or underestimate the stiffness (see Figure 1), limiting the fidelity of associated FEA simulations. A reduced set of the experimental data, bounded by the maximum expected strain, may mitigate these issues. However, to decide *a priori* the expected strain can be challenging. Increasing the order of the material model represents another method to reduce fitting errors, however, this can be potentially harmful to the robustness and the stability of the simulation (Schumacher et al., 2020). Previous work has tried to improve the stability and performance of the modelling by using FEA simulation to fit the model parameters (Hartmann et al., 2003; Fu et al., 2013; Schumacher et al., 2020; Hartmann and Gilbert, 2021). The main idea behind this approach is to find the parameters of the material model by solving an inverse optimization problem. This system explores the search space in order to minimize the error between the finite element analysis and the experimentally obtained data. While these techniques may be useful for large deformations; the potential disparity between local strains in the gauge and the globally measured strains still exists.

To address these issues, we propose Evolutionary Inverse Material Identification (EIMI), a material characterization approach aimed at identifying the parameters for a material based on the target application, in our case: MASRs. We aim to characterize materials by using a simulated representation actuated using the same method as used during experiments. Having defined a simulation model, we can then create, deform and observe an equivalent specimen of soft material in a real, controlled environment. Finally, we define an optimization problem where the parameters of the material model comprise the search space while the error between the experimental data and the simulation is minimized. The problem defined is often non-linear and gradient methods may fail to find an optimal solution. We address this issue by using, for the first time, an optimizer based on an evolutionary strategy. An evolutionary algorithm (EA) allows a good balance between exploitation and exploration of the search space (Neri and Tirronen, 2010; Deb, 2014). This technique has several benefits, first, using a simulation model to search the parameters for the material allows us to obtain a result that guarantees robustness and increases the performance of the simulation. Second, by using the same actuation method in the simulation and experiment, our resulting material model will be optimized for the target application. This is because EIMI will intrinsically reduce the error between the observed stress and the predicted stress from the analytical model. Moreover, using EIMI over the conventional tensile testing approach may solve the problem of heterogeneous deformation encountered by the specimen.

In the next section, our framework for material characterization is presented. First, we define the FEA model and the fabrication steps to create the equivalent experimental sample. Next, using the proposed comparison metric, we discuss the steps to minimize the fitting errors using an EA. To demonstrate our characterization approach, we focus on a MASR, made using two soft polymers commonly found in soft robotics applications: Ecoflex™ 00-50 and Dragon Skin™ 10 Medium. Finally, in the *Experimental Evaluation* section the performances of the models are characterized using the framework presented, and the resulting model parameters are compared with the model parameters obtained using the conventional fitting approach.

## Characterization Framework

**FIGURE 2:**
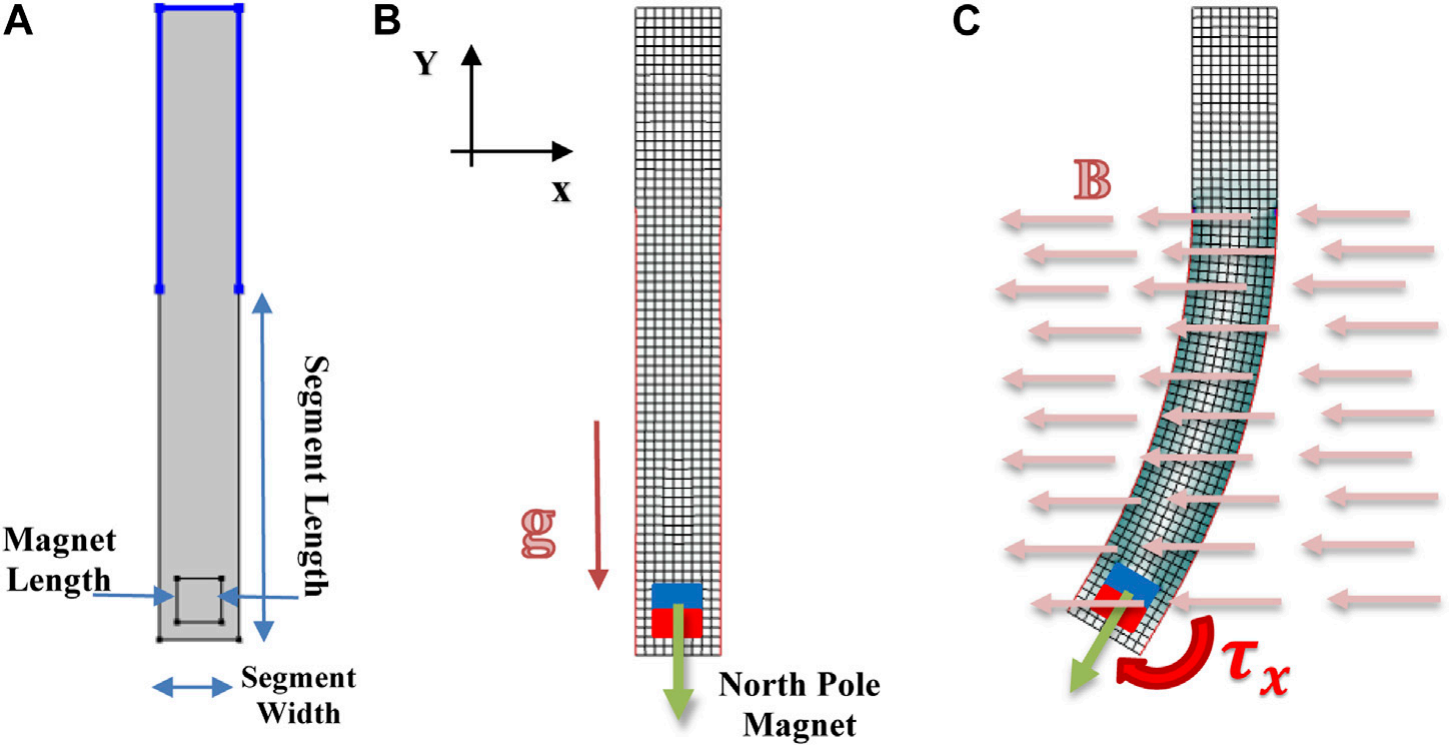
Simulated representation: **(A)** geometry of the sample is shown; **(B)** mesh used for the FEM simulation, the direction of the magnetic field, as well as the magnetization direction M, are shown; **(C)** an example simulation result is also shown.

To demonstrate our characterization approach, we first developed a FEA model of a simple MASR. The design takes the form of a rectangular body with a small permanent magnet embedded at the distal end, as shown in Figure 2. The body of the MASR bends in response to a magnetic torque generated from the interaction between the embedded magnet and a globally applied magnetic field. We defined a procedure to allow comparison between the FE simulation and the experimental data; thus, developing an objective function for the optimization problem. Comprehensive details of our approach are presented in the following sections.

### FEA Model

As previously mentioned, conventional uni-axial mechanical tests on soft materials deform standardized samples over several times their original length (Marechal et al., 2020). However, in more realistic soft robotic applications, especially those driven *via* magnetic fields, the material may experience only a fraction of these deformations. To improve fidelity between testing for characterization and application, we propose simulating the application-specific actuation method (i.e., magnetically induced forces and torques) within the FEA simulation and solving an optimization problem using an EA to characterize the material. Due to the nature of the EA, the simulation model may be invoked many times, with the potential to significantly increase computational time. Therefore, we developed our FEA model with a balance between accuracy and computational cost to simulate the magneto-mechanical response of our soft robotic designs. This combines analytical mechanical models used to predict nonlinear elastic behavior of polymers with magnetic interaction forces and torques based on a dipole approximation (Petruska and Abbott, 2013; Abbott et al., 2020).

For the mechanical model, we consider soft materials to be homogenous and isotropic. In previous work, it has been shown that compressibility and viscoelasticity may be neglected for lower strains (Steck et al., 2019). In general, to represent the deformed configuration χ of a soft material the following Cauchy tensor is used (Wang et al., 2020):

$$\sigma =J^{-1}\frac{\partial W}{\partial F}F^{T},$$ (1)

where F = ∇χ is the deformation gradient of the deformed configuration χ , J=det F is the volumetric Jacobian of the deformation and W is the strain energy function that characterizes the specific material. If the material is considered incompressible the Jacobian will be equal to 1. Many different energy functions have been proposed to characterize the behavior of soft hyperelastic materials (Mihai and Goriely, 2017). For this work, we focus on models that have been shown to work well for lower strains (Steck et al., 2019): Neo Hookean, Mooney-Rivlin, Yeoh and Ogden of the third order. These material models, up to the third order, give a wide search space whilst also testing the fitting capability of the framework. The functions considered in the characterization framework are presented in Table 1 in terms of the principal stretches ${\text{λ}}_{1},{\text{λ}}_{2},{\text{λ}}_{3}$ . The energy functions each contain a series of constant parameters C_i_ and α_i_ that define the specific material. These parameters can be tuned based on experimental observations. These parameters, C_i_ and α_i_, thus constitute the decision space of the optimization algorithm.

**TABLE 1:** Strain Energy functions for incompressible hyperelastic materials (Mihai and Goriely, 2017).

Model	Strain energy function
Neo Hookean	$W=\frac{C_{1}}{2}\left(\lambda_{1}^{2}+\lambda_{2}^{2}+\lambda_{3}^{2}-\;3\right)$
Mooney–Rivlin	$W=C_{10}\left(\lambda_{1}^{2}+\lambda_{2}^{2}+\lambda_{3}^{2}-\;3\right)+C_{01}\left(\lambda_{1}^{-2}+\lambda_{2}^{-2}+\lambda_{3}^{-2}-\;3\right)$
Yeoh	$W=\overset{3}{\underset{i=1}{\sum }}\frac{{\text{C}}_{\text{i}}}{2\text{i}}{\left(\lambda_{1}^{2}+\lambda_{2}^{2}+\lambda_{3}^{2}-\;3\right)}^{i}$
Ogden	$W=\overset{3}{\underset{i=1}{\sum }}\frac{{\text{C}}_{\text{i}}}{2\alpha_{i}^{2}}\left(\lambda_{1}^{2_{\mathrm{\alpha 1}}}+\lambda_{2}^{2_{\mathrm{\alpha 1}}}+\lambda_{3}^{2_{\mathrm{\alpha 1}}}-\;3\right)$

The soft material of the FE simulation is then deformed through the interaction with a rigid body, in our case a permanent magnet. The forces ***f*** and torques τ on the permanent magnet are induced *via* a homogeneous magnetic field. These can be modeled by employing the following equations (Jeon et al., 2019; Abbott et al., 2020):

f=∇(B⋅m), (2)

τ = m × B, (3)

where m  is the magnetic moment of the permanent magnet, B  is the magnetic flux density. In a uniform magnetic field, magnetic forces are zero as the field gradient is ∇B=0 **,** leaving only the restoring torque to act on the permanent magnet. In addition, the magnetization of an object is defined as m=vχ(H)H where v is the volume of the sample, χ(H) is the susceptibility tensor, H  is the applied magnetic field where $H=B/{\text{μ}}_{0}$ defines the relationship between the magnetic field and magnetic flux density in which ${\text{μ}}_{0}\;$ is the magnetic permeability of free space. In our case, we can simplify these equations without undermining the validity of the model with the following assumptions. First, since we are using a permanent magnet subject to a small magnetic field, this has no influence on the magnetic moment. Second, since the permanent magnet is physically small, it can be approximated as a point dipole by neglecting its geometry and therefore the susceptibility tensor becomes an identity matrix. As a result, the magnetic moment can be simplified as:

$$m=v\frac{B}{{\text{μ}}_{0}}=vM,$$ (4)

A global coordinate system can be established since the magnetic actuation is the result of an external magnetic field interacting with the magnet. We consider actuation within a 2D plane, therefore, only one component of the magnetic field is non-zero. We define this to be the x -axis according to our reference frame ( $B_{x}\neq 0$ and $B_{y},B_{z}=0$ ). The SR was fixed at one end and constrained to bend in only one direction by the applied magnetic field, constraining the magnetic moment to the xy plane. The simplified relationship can be seen in Eq. 5.

$$\tau_{z}=-m_{y}B_{x}=-|m||B|\sin{\text{θ}}_{z},$$ (5)

Finally (see *Sample Fabrication and Experimental Setup* section), body forces due to gravity were integrated in the simulation model, to reconcile with the experimental setup.

The geometry and constraints of the MASR were selected to allow reduction of the model to a 2D plane (Figure 2A), and thus to perform 2D simulations with computational efficiency, by considerably reducing the nodes of the mesh and therefore the complexity of the system. The model so defined can be assumed as plane strain, since the cross-sectional thickness is sufficiently large to consider the depth dimension of the strain tensor equal to zero. For each simulation, geometry of the design was varied using 3 parameters: the length and the width of the robot, and the length of the magnet to match the physical dimensions of the MASR. A quadrilateral mesh was then generated based on the planar geometry (see Figure 2B), and the mesh size was determined after a dependency analysis over the magnet displacement for a fixed magnetic flux density of 15 mT. The magnet was positioned within the robot body with the magnetization direction (north pole) oriented towards the distal end. Therefore, the magnetization direction of the magnet and the externally applied field are perpendicular when the sample is in the resting position, thus maximizing torque (see Figure 2B). The simulation model response may then be evaluated for a range of magnetic fields (Figures 2C, 5B), in line with experimental testing. The model was implemented using COMSOL Multiphysics^®.^ V5.4 (COMSOL, Sweden) and solved using Multifrontal Massively Parallel Sparse direct Solver. The number of quad mesh elements used for each geometry are summarized in Table 2.

**TABLE 2:** MASR geometry and number of quad mesh elements used for each type of sample.

	Segment		Magnet	Mesh elements
	Length (mm)	Width (mm)	Length (in)	
Type 1	25	5.67	1/16	648
Type 2	35	5.67	1/16	759
Type 3	35	8.5	3/16	783
Type 4	45	8.5	3/16	913

### Sample Fabrication and Experimental Setup

To provide experimental data for the optimization, samples were prepared and tested under a varying magnetic field. Four versions of the design were prepared with the geometrical parameters summarized in Figure 2. One of these geometries (Type 1) was used for training the material characterization through the optimization algorithm, while the others were used for validation (Type 2, 3, 4).

**FIGURE 3:**
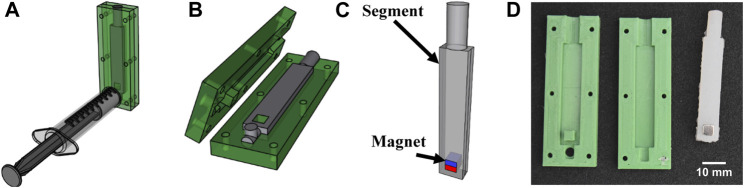
Fabrication steps for magnetic robots are shown: **(A)** injection of the silicone in the negative mold; **(B)** curing of the silicone; **(C)** magnet insertion **(D)** image of the mold and sample.

Molds were created for each geometry using a 3D Printer (Ultimaker S5), as shown in Figure 3. Samples were created using two soft elastomers: Dragon Skin™ 10 Medium and Ecoflex™ 00-50 (Smooth-on Inc., United States). For each material, the two-part components were mixed in equal weight using a high vacuum-mixer (Arv-10 from THINKYMIXER, Japan), at a pressure of 20 kPa for 90 s with the centrifuge set at 1,400 rpm to thoroughly mix and remove any air bubbles. A volume of material equal to two times the size of the specimen was slowly injected from the bottom of the mold and residual bubbles and excessive material were expelled using a port at the top of the mold. The material was allowed to cure at room temperature as specified by the manufacturer (5 h for Dragon Skin™ 10 Medium and 3 h for Ecoflex™ 00-50). Once cured, the samples were demolded and the appropriately sized magnets (grade N52, K&J Magnetics Inc., United States) were inserted in the pre-allocated space, with the magnetization direction (north pole) pointing towards the distal end of the MASR.

**FIGURE 4:**
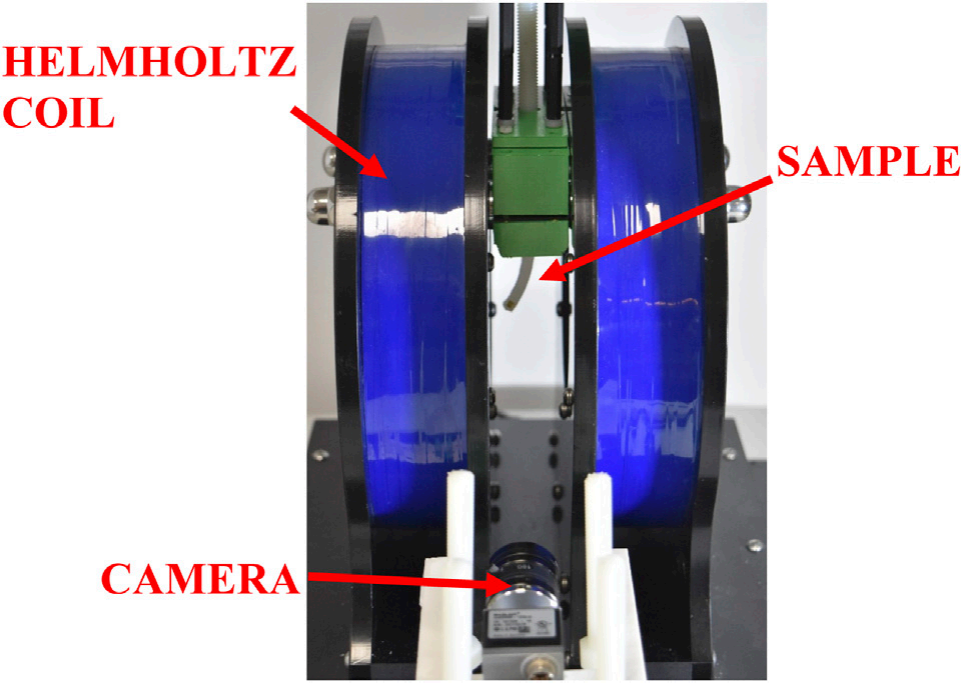
Experimental setup for testing magnetic robots. The sample is mounted in the center of the Helmholtz Coil and the deflection is captured using a side-view camera.

As shown in Figure 4, samples were tested using a uniform magnetic field, generated using a 1D Helmholtz Coil (DXHC10-200, Dexing Magnet Tech. Co., Ltd., Xiamen, China). During the experiment, the current in the coil was increased in discrete amounts. As a result, the magnetic field was increased proportionally to the current in the coil as described by Eq. 6 (Abbott et al., 2020).

$$B_{x}={\left(\frac{4}{5}\right)}^{\left(3/2\right)}\frac{{\text{μ}}_{0}nI}{R},$$ (6)

where n is the number of coil windings, I is the current in the coils and R is the radius of the coils. For each discrete current step, the sample deformation was recorded using a camera (acA 2040-120um, Basler AG, Germany) and post-processed (Figure 4). The resolution of the images was 0.043 px/mm.

**FIGURE 5:**
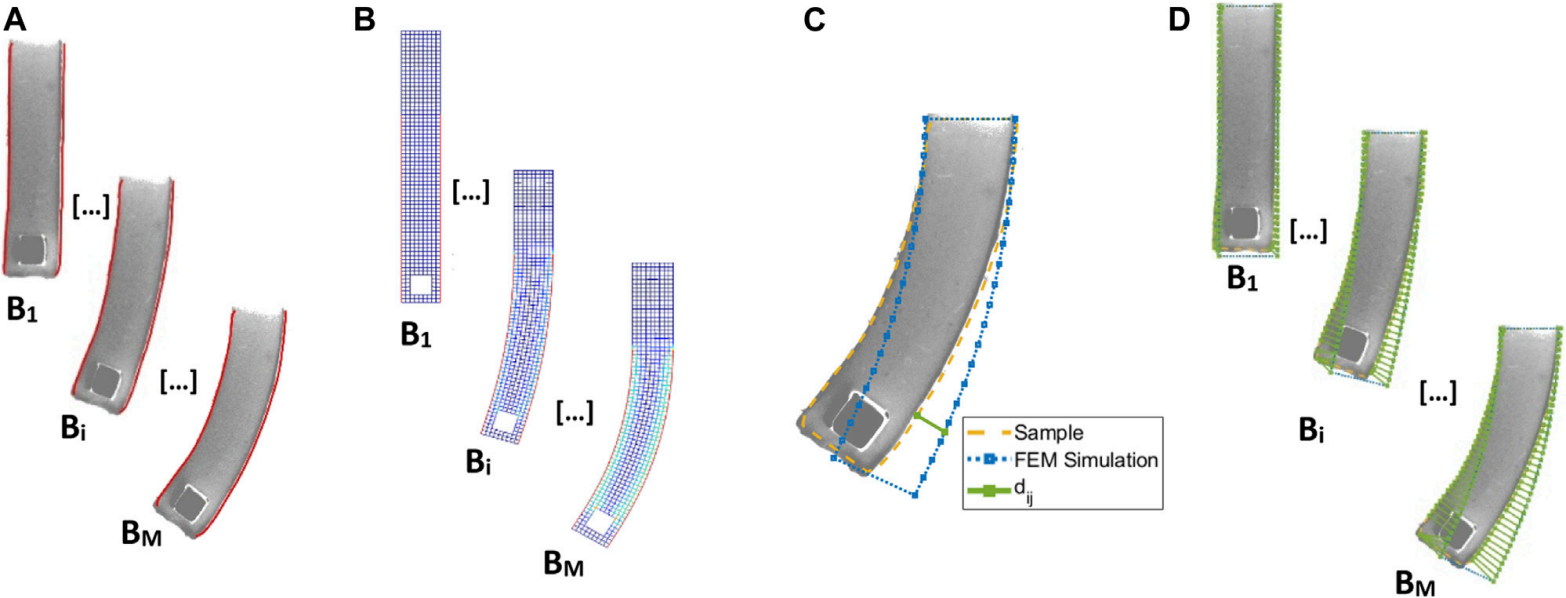
The steps used to obtain the distance between the FE simulation and the experimental data (see Eq. 10): **(A)** image segmentation and feature extraction of the left and right edge of the robot; **(B)** FEM simulation for a chosen model and parameters $\;p=\left[C_{1}\ldots C_{N},\alpha_{1}\ldots \alpha_{M}\right];$ **(C)** distance evaluation *d*_*ij*_ (green line) for each point j of the FEM simulation from the experimental data; **(D)** evaluation of the variance for each value of magnetic flux density *B*_i_.

To extract image features intrinsically rich in information that could be used to compare the experimental deformation with the simulation model, we developed a post-processing procedure to measure the outer edges of the sample. Bespoke image processing code was developed using MATLAB (Image Processing Toolbox, MathWorks, United States), to allow segmentation and edge extraction where the deflection/deformation is more prominent along the longitudinal length of the sample. The following steps were followed: 1) high contrast images were obtained; allowing the sample edges to be easily extracted; 2) the boundary of the object was extracted, and the corners of the sample were determined from the edges; and 3) after splitting the edge of the sample into different segments, the relevant ones were saved to be used as a target in the characterization process (see Figure 5A).

### Benchmark Functions

After the simulated and experimental results were obtained, a metric was needed to compare the two and allow for optimization. We propose the following approach to solve the problem where for each value of the magnetic flux density ( $B_{i}$ ) tested, the predicted simulation model and the recorded experimental data can be compared. First, the distances (or error) $d_{ji}$ between each point j of the simulation contour and corresponding closest point of the experimental sample were evaluated (see Figure 5C). The number of points were in direct correlation with the mesh, such that from geometry Types 1 to 4, the number of points j considered were 80, 110, 70, and 100 respectively. The set of distances created have high dimensionality and thus are impractical to use. Therefore, we used a statistical method to evaluate the fitting errors between the FEA model and the experimental data. The standard error $SE_{i}$ is evaluated for each equilibrium state ( i ) for the magnetic field $B_{i}$ and the chosen model parameters $p=\left[C_{1}\ldots C_{N},{\text{α}}_{1}\ldots {\text{α}}_{M}\right]$ used for the simulation, such as:

$$\mu_{di}\left(p,B_{i}\right)=\frac{1}{N_{i}}\underset{j}{\sum }d_{ji}\left(p,B_{i}\right),$$ (7)

$$\sigma_{di}\left(p,B_{i}\right)=\sqrt{\frac{1}{N_{i}}\underset{j}{\sum }{\left(d_{ji}\left(p,B_{i}\right)-\mu_{di}\left(p,B_{i}\right)\right)}^{2}},$$ (8)

$$SE_{i}\left(p,B_{i}\right)=\frac{\sigma_{d}\left(p,B_{i}\right)}{\sqrt{N_{i}}},$$ (9)

where $\mu_{di}$ and $\sigma_{di}$ are, respectively, the mean and the standard deviation of the distances $d_{ji}$ , and $N_{i}$ is the number of points defining the contours of the simulation model for equilibrium state i . After evaluating the standard error for the equilibrium state, we obtained a set of M data (see Figure 5D). Changing parameters for a hyperelastic model (or the model type) may result in a stiffer or softer material compared to the experimental data. This translates into either smaller or larger deflections, making the simulation model drift from the experimental data. To further reduce the dimensionality of this minimization, we decided to evaluate the mean of the standard error ( $\mu_{SE}$ ) across all equilibrium test conditions:

$$\mu_{SE}\left(p\right)=\frac{1}{M}\underset{i}{\sum }SE_{i}\left(p,B_{i}\right),$$ (10)

The metric in Eq. 10 was used to compare the different material models and as an objective function for the parameter fitting. In addition, the standard deviation ( $\sigma_{SE}$ ) of the standard error was also used for comparison purposes in the experimental evaluation section (see *Experimental Evaluation* section):

$$\sigma_{SE}\left(p\right)=\frac{1}{M}\underset{i}{\sum }{\left(SE_{i}\left(p,B_{i}\right)-\mu_{SE}\left(p\right)\right)}^{2},$$ (11)

### Algorithm Development and Application

Here, we defined an optimization problem that allows fitting of the material parameters for a specific analytical model (Table 1). With the goal of minimizing the error by varying the parameters p  of the hyperelastic model in the search space, the following minimization problem was defined:

$$\begin{array}{cc} \underset{x}{\text{min}} & \mu_{SE}\left(p\right)\\ \text{subject to } & p_{l}\leq p\leq p_{u} \end{array},$$ (12)

where the parameters of the hyperelastic models are subject to upper and lower bounds based on physical constraints. The dimension of p is naturally connected to the order of the model selected. To reduce the search space for p we upper bound the vector element of p to 1 MPa. The objective function involves computing the simulation model using the parameters p and then comparing these with the experimental results using Eq. 10. To solve the defined problem, conventional approaches often rely on the use of simple relations that can be solved using gradient methods such as the Levenberg-Marquardt algorithm (Hartmann et al., 2003; Schumacher et al., 2020). However, due to the complexity of the simulation model, these methods are infeasible for our approach since they fail to navigate over a complex objective landscape. Therefore, we opted to use an EA to perform the optimization.

**FIGURE 6:**
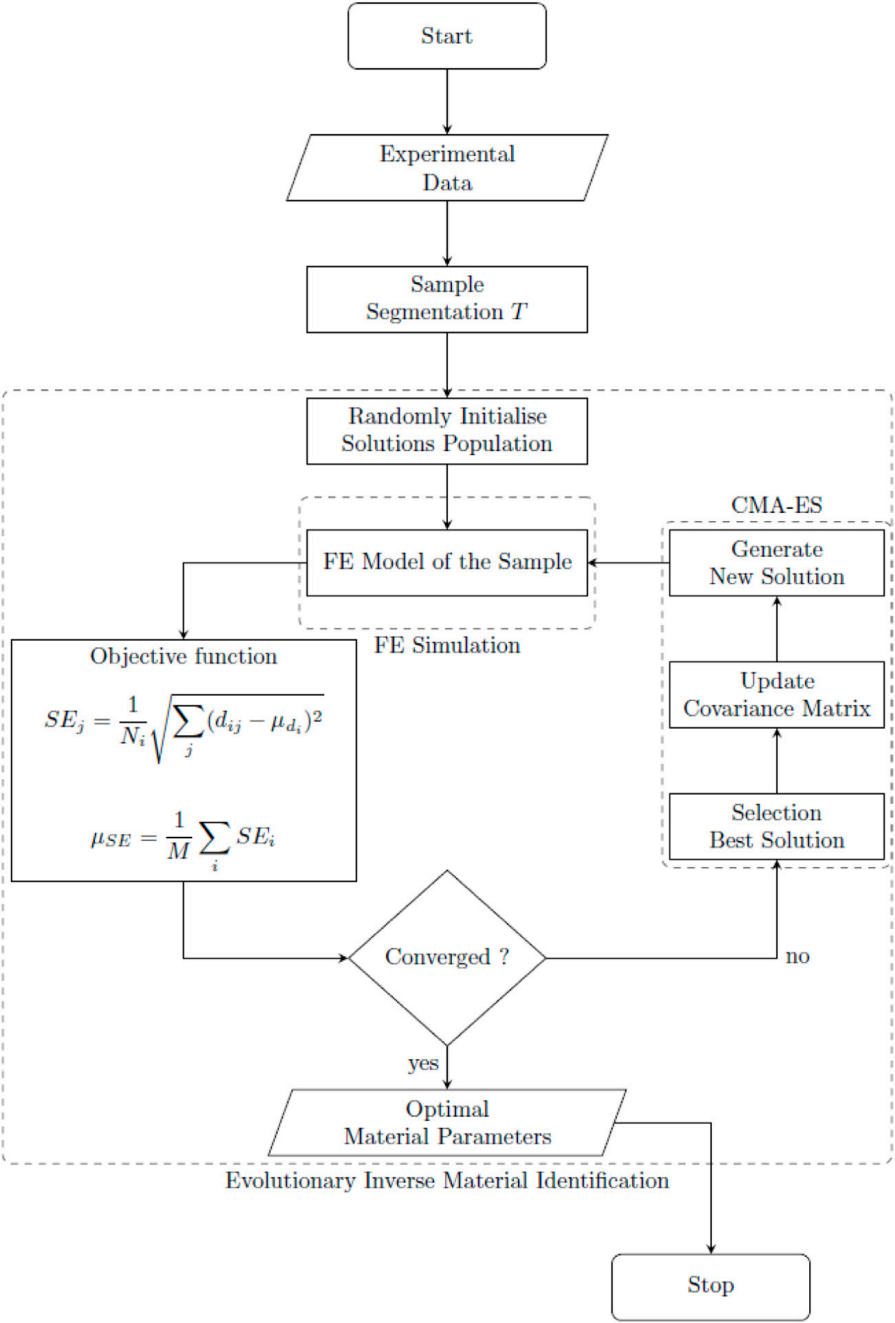
Evolutionary Inverse Material Identification (EIMI) flowchart.

EAs are, in general, based on some stochastic phenomena. This allows a random walk in the search space to improve the exploration features. Furthermore, these types of algorithms do not require complete information about the problem landscape. These characteristics made this type of algorithm an ideal solution for the problem defined here. In general, EAs are defined as a cyclic process with specific steps (see Figure 6). Initially, a random solution or set of solutions is created, then a two-step process is repeated for several generations (cycles) until the result converges. The first step involves a selection process where the best model parameters are chosen based on the metric (Eq. 10). These solutions are then used to generate new solutions through a stochastic procedure that will replace the solution discarded in the previous step, The stochastic procedure for generating newer solutions is partially based on the information contained in the solution that survived the selection phase (Deb, 2014).

In our framework, solutions that don’t comply with Drucker’s Stability are immediately discarded in the selection phase, specifically, any result that did not converge to a residual error $1\text{x}10^{-4}$ within 25 Newton Generation. These results do not represent a valid solution for our fitness function.

Several EA strategies have been proposed, however, one of the most successful to date has been the Covariance Matrix Adaptation Evolution Strategy (CMA-ES) (Hansen and Ostermeier, 2001). This algorithm has shown great potential for solving complex problems compared to other optimization strategies (Caraffini et al., 2013, 2019). This search strategy selects the best solution ν in the population of η individuals for each cycle. It then uses the distribution of the new population to incrementally update a covariance matrix. The principal components of the matrix are subsequently used to direct the exploration in the search space and generate new solutions. By updating the covariance matrix incrementally, the search leads to solutions that show an improvement compared to previous cycles. This population-based strategy is a good compromise between the exploration of the search space and the exploitation of the best solution. For this reason, we decided to use the CMA-ES as a solver for EIMI. Five parameters need to be defined, with the necessary inputs for the algorithm shown in Table 3. The steps described to reach material parameters are shown in the flowchart in Figure 6. The process was implemented in MATLAB (Matlabworks, United States), with a connection to the FE simulation in COMSOL Multiphysics^®^ V5.4 using the plugin LiveLink™.

**TABLE 3:** CMA-ES parameters.

Name parameter	Value	Note
N	Number of Parameters	Dimension of the decision space
η	4 + 3logN	Dimension of the population
ν	η/2	Solutions used to generate newer solutions
Step Size	0.3	Step Size, determine the speed of convergence
Tolerance	$10^{-6}$	Stop criteria for the algorithm

## Experimental Evaluation

We characterized two elastomers: Dragon Skin™ 10 Medium and Ecoflex™ 00-50 (Smooth-On Inc, United States). Four samples for each material were created using the dimensions listed in Table 2. The samples were magnetically actuated in the Helmholtz coil, by increasing the current in the coils incrementally from 0 to 1.5 A in steps of 0.1 A for Dragon Skin™ 10 Medium and from 0 to 1 A in steps of 0.1 A for Ecoflex™ 00-50. The current can be translated to magnetic flux density with a conversion factor of 4.7 mT/A. The deformation at each current step was recorded, and the samples were segmented (Figure 5). The resulting database was then split based on geometry. The samples with the Type 1 geometry (Table 2) were used for the material fitting while the rest were used for validation.

### EIMI Analysis

The data from the samples chosen for the material characterization are used to define the optimization problem (see *Benchmark Functions* section) to solve the CMA-ES. The number of evaluations and generations required by the CMA-ES to converge to an optimal solution and obtain a set of model parameters are shown in Table 4. With an increasing number of parameters in the material model, the table displays the growth in the number of generations that were required to converge. This is expected, as they represent a complex objective landscape that is more difficult to explore. The table also shows an outlier, due to the stochastic nature of the EA. The characterization of Ecoflex™ 00-50 using the Ogden model required only 8 generations, despite the search space having high dimensionality with 6 model parameters.

**TABLE 4:** Number of function evaluations and generations required by the CMA-ES to characterize the materials.

	Dragon Skin™ 10 medium		Ecoflex™ 00-50	
	N Evaluation	N Generation	N Evaluation	N Generation
Neo Hookean	91	43	97	46
Mooney–Rivlin	173	55	299	97
Yeoh	398	130	341	111
Ogden	621	153	81	8

The parameters for the models obtained using EIMI are shown respectively on the top halves of Table 5 for Dragon Skin™ 10 Medium and Table 6 for Ecoflex™ 00-50. The two tables list the mean of the standard error and mean of the standard deviation using Eqs 10, 11 for each type of geometry and model using both our approach and the conventional approach (detailed below).

**TABLE 5:** Results for Dragon Skin™ 10 Medium.

	Model	$\varepsilon_{\mathrm{eng}}$	Parameters	Type 1^1^		Type 2		Type 3		Type 4			
				${µ}_{\mathrm{SE}}$ ^2^	$\sigma_{\mathrm{SE}}$ ^2^	${µ}_{\mathrm{SE}}$ ^2^	$\sigma_{\mathrm{SE}}$ ^2^	${µ}_{\mathrm{SE}}$ ^2^	$\sigma_{\mathrm{SE}}$ ^2^	${µ}_{\mathrm{SE}}$ ^2^	$\sigma_{\mathrm{SE}}$ ^2^	${µ}_{\mathrm{SE}}$ ^2^	$\sigma_{\mathrm{SE}}$ ^2^
Method Proposed	Neo Hookean		μ = 4.317 × 10⁴ Pa	0.220	0.041	**0.403**	0.027	0.440	0.039	1.131	0.376	0.549	0.400
	Mooney Rivlin		C₀₁ = 1.190 × 10⁵ Pa, C₁₀ = −9.740 × 10⁴ Pa	0.219	0.040	0.405	0.028	**0.430**	0.027	1.140	0.378	0.549	0.405
	Yeoh		C₁ = 2.406 × 10⁴ Pa, C₂ = −1.707 × 10⁵ Pa, C₃ = 2.055 × 10⁶ Pa	**0.211**	0.033	0.421	0.037	0.447	0.036	1.177	0.329	0.564	0.422
	Ogden		C₁ = 1.362 × 10⁴ Pa, C₂ = −5.762 × 10⁵ Pa, C₃ = 7.633 × 10⁵ Pa α₁ = 9.330, α₂ = 3.376, α₃ = 2.487	0.241	0.045	0.444	0.017	0.443	0.028	**0.997**	0.323	**0.531**	0.325
Conventional	Neo Hookean	[0.0.2]	μ = 2.040 × 10⁴ Pa	1.356	0.473	1.505	0.467	1.870	0.617	1.170	0.272	1.475	0.297
		[0.0.5]	μ = 2.319 × 10⁴ Pa	1.129	0.387	1.305	0.399	1.565	0.510	0.929	0.184	1.232	0.270
		[0,1]	μ = 2.818 × 10⁴ Pa	0.783	0.243	0.977	0.272	1.095	0.325	0.628	0.091	0.871	0.207
		[0,10.4]	μ = 7.526 × 10⁴ Pa	0.878	0.428	1.251	0.400	1.474	0.597	2.581	1.038	1.546	0.732
	Mooney Rivlin	[0.0.2]	C₀₁ = 6.900 × 10⁴ Pa, C₁₀ = −5.549 × 10⁴ Pa	/	/	1.054	0.303	/	/	0.672	0.097	0.863	0.270
		[0.0.5]	C₀₁ = 4.068 × 10⁴ Pa, C₁₀ = −2.355 × 10⁴ Pa	0.454	0.096	0.650	0.123	0.660	0.128	0.659	0.183	0.606	0.101
		[0,1]	C₀₁ = 4.908 × 10⁴ Pa, C₁₀ = −3.511 × 10⁴ Pa	0.804	0.252	0.996	0.279	1.126	0.338	0.640	0.091	0.892	0.214
		[0,10.4]	C₀₁ = 9.272 × 10⁴ Pa, C₁₀ = −1.357 × 10⁵ Pa	3.635	2.035	6.381	3.161	6.066	3.159	9.303	4.533	6.346	2.322
	Yeoh	[0.0.2]	C₁ = 1.302 × 10⁴ Pa, C₂ = 1.249 × 10⁵ Pa, C₃ = −5.947 × 10⁵ Pa	/	/	0.909	0.215	/	/	**0.603**	0.093	0.756	0.216
		[0.0.5]	C₁ = 1.953 × 10⁴ Pa, C₂ = 7.410 × 10³ Pa, C₃ = −3.117 × 10³ Pa	0.258	0.047	0.463	0.025	0.446	0.027	0.922	0.297	**0.522**	0.282
		[0,1]	C₁ = 2.045 × 10⁴ Pa, C₂ = 4.473 × 10³ Pa, C₃ = −5.341 × 10² Pa	**0.227**	0.044	**0.425**	0.018	**0.429**	0.029	1.022	0.334	0.526	0.344
		[0,10.4]	C₁ = 4.873 × 10⁴ Pa, C₂ = 3.171 × 10² Pa, C₃ = −1.044 Pa	1.183	0.608	1.724	0.651	1.966	0.868	3.235	1.365	2.027	0.869
	Ogden	[0.0.2]	C₁ = −6.831 × 10⁶ Pa, C₂ = 4.031 Pa, C₃ = −6.826 Pa	2.622	1.477	4.356	2.155	4.377	2.267	6.737	3.290	4.523	1.690
			α₁ = 1.476 × 10⁻¹, α₂ = 5.027 × 10⁻¹, α₃ = 1.454 × 10⁻¹										
		[0.0.5]	C₁ = −2.323 × 10⁷ Pa, C₂ = 1.793 × 10⁻⁶ Pa, C₃ = 7.254 Pa	2.487	1.396	4.088	2.003	4.146	2.133	6.387	3.096	4.277	1.603
			α₁ = 9.959 × 10⁻², α₂ = 2.011 × 10¹, α₃ = 3.233 × 10⁻¹										
		[0,1]	C₁ = −5.740 × 10⁵ Pa, C₂ = 1.333 × 10⁻¹ Pa, C₃ = 1.329 × 10⁻² Pa	3.869	2.157	6.742	3.316	6.463	3.352	9.826	4.757	6.725	2.439
			α₁ = 2.702 × 10⁻¹, α₂ = 1.034, α₃ = 4.038										
		[0,10.4]	C₁ = −9.193 × 10⁴ Pa, C₂ = 1.641 × 10⁻¹ Pa, C₃ = −7.673 × 10⁻² Pa	3.342	1.887	5.761	2.894	5.592	2.934	8.562	4.226	5.814	2.138
			α₁ = 2.696, α₂ = 3.402, α₃ = 3.576										

1 Sample used for the identification for the approach proposed.

2 The value dimension is in (mm).

The best result for each methodology is shown in bold.

**TABLE 6:** Results for Ecoflex™ 00-50. The best result for each methodology is shown in bold.

	Model	$\varepsilon_{\mathrm{eng}}$	Parameters	Type 1^1^		Type 2		Type 3		Type 4			
				${µ}_{\mathrm{SE}}$ ^2^	$\sigma_{\mathrm{SE}}$ ^2^	${µ}_{\mathrm{SE}}$ ^2^	$\sigma_{\mathrm{SE}}$ ^2^	${µ}_{\mathrm{SE}}$ ^2^	$\sigma_{\mathrm{SE}}$ ^2^	${µ}_{\mathrm{SE}}$ ^2^	$\sigma_{\mathrm{SE}}$ ^2^	${µ}_{\mathrm{SE}}$ ^2^	$\sigma_{\mathrm{SE}}$ ^2^
Method Proposed	Neo Hookean		μ = 2.684 × 10⁴ Pa	**0.276**	0.034	0.541	0.038	0.655	0.160	0.668	0.234	0.535	0.182
	Mooney Rivlin		C₀₁ = −5.099 × 10⁴ Pa, C₁₀ = 6.463 × 10⁴ Pa	**0.276**	0.033	0.556	0.033	0.677	0.172	0.689	0.247	0.550	0.192
	Yeoh		C₁ = 1.398 × 10⁴ Pa, C₂ = −3.195 × 10⁴ Pa, C₃ = 4.480 × 10⁵ Pa	0.277	0.033	0.564	0.035	0.682	0.167	0.705	0.251	0.557	0.197
	Ogden		C₁ = −2.728 × 10³ Pa, C₂ = −1.651 × 10⁵ Pa, C₃ = 4.920 × 10⁵ Pa α₁ = −1.719, α₂ = −2.883 × 10⁻¹, α₃ = −3.947 × 10⁻³	0.324	0.032	**0.518**	0.051	**0.611**	0.119	**0.617**	0.191	**0.518**	0.137
Conventional	Neo Hookean	[0.0.2]	μ = 9.175 × 10³ Pa	1.744	0.594	1.280	0.357	1.815	0.663	1.391	0.524	1.558	0.262
		[0.0.5]	μ = 1.039 × 10⁴ Pa	1.588	0.545	1.202	0.331	1.619	0.588	1.256	0.464	1.416	0.218
		[0,1]	μ = 1.118 × 10⁴ Pa	1.492	0.513	1.147	0.312	1.499	0.540	1.171	0.424	1.327	0.195
		[0,16.4]	μ = 4.354 × 10⁴ Pa	0.791	0.315	1.193	0.309	1.343	0.605	1.332	0.677	1.165	0.258
	Mooney Rivlin	[0.0.2]	C₀₁ = 5.516 × 10⁴ Pa, C₁₀ = −5.249 × 10⁴ Pa	/	/	/	/	/	/	/	/	/	/
		[0.0.5]	C₀₁ = 1.636 × 10⁴ Pa, C₁₀ = −8.039 × 10³ Pa	0.905	0.286	0.767	0.147	0.830	0.227	0.683	0.164	0.796	0.094
		[0,1]	C₀₁ = 1.447 × 10⁴ Pa, C₁₀ = −5.539 × 10³ Pa	0.794	0.236	0.693	0.108	0.725	0.171	0.611	0.125	0.706	0.076
		[0,16.4]	C₀₁ = 6.233 × 10⁴ Pa, C₁₀ = −2.203 × 10⁵ Pa	2.826	1.559	4.036	2.026	3.470	1.988	3.641	2.236	3.493	0.504
	Yeoh	[0.0.2]	C₁ = 1.768 × 10³ Pa, C₂ = 1.289 × 10⁵ Pa, C₃ = −6.313 × 10⁵ Pa	/	/	1.509	0.414	/	/	1.538	0.529	1.524	0.021
		[0.0.5]	C₁ = 8.603 × 10³ Pa, C₂ = 5.213 × 10³ Pa, C₃ = −4.209 × 10³ Pa	0.829	0.246	0.728	0.124	0.761	0.182	0.644	0.140	0.741	0.077
		[0,1]	C₁ = 9.800 × 10³ Pa, C₂ = 9.536 × 10² Pa, C₃ = −1.710 × 10² Pa	0.650	0.168	0.606	0.061	**0.615**	0.109	**0.546**	0.101	0.604	0.043
		[0,16.4]	C₁ = 1.385 × 10⁴ Pa, C₂ = 1.110 × 10² Pa, C₃ = −8.767 × 10⁻² Pa	**0.279**	0.030	**0.572**	0.027	0.703	0.189	0.713	0.263	**0.567**	0.202
	Ogden	[0.0.2]	C₁ = −6.190 × 10^6^ Pa, C₂ = −6.405 Pa, C₃ = 3.679 Pa	2.831	1.558	4.004	1.998	3.496	1.998	3.634	2.227	3.491	0.490
			α₁ = 1.339 × 10⁻¹, α₂ = 1.331 × 10⁻¹, α₃ = 4.581 × 10⁻¹										
		[0.0.5]	C₁ = −1.263 × 10⁷ Pa, C₂ = 2.298 Pa, C₃ = 1.807 Pa	2.758	1.513	3.889	1.929	3.418	1.947	3.541	2.164	3.402	0.473
			α₁ = 9.804 × 10⁻², α₂ = 3.050 × 10⁻¹, α₃ = 3.050 × 10⁻¹										
		[0,1]	C₁ = 5.433 × 10⁶ Pa, C₂ = 1.923 × 10⁴ Pa, C₃ = −1.754 × 10¹ Pa	2.473	1.338	3.451	1.658	3.119	1.752	3.193	1.928	3.059	0.416
			α₁ = 2.447 × 10⁻¹, α₂ = 6.933, α₃ = 7.507 × 10⁻²										
		[0,16.4]	C₁ = 7.274 × 10^5^ Pa, C₂ = −1.385 Pa, C₃ = 6.724 × 10⁻¹ Pa	2.532	1.374	3.540	1.714	3.180	1.792	3.264	1.976	3.129	0.427
			α₁ = 3.210, α_2_ = 3.295, α₃ = 3.372										

1 Sample used for the identification for the approach proposed.

2 The value dimension is in (mm).

**FIGURE 7:**
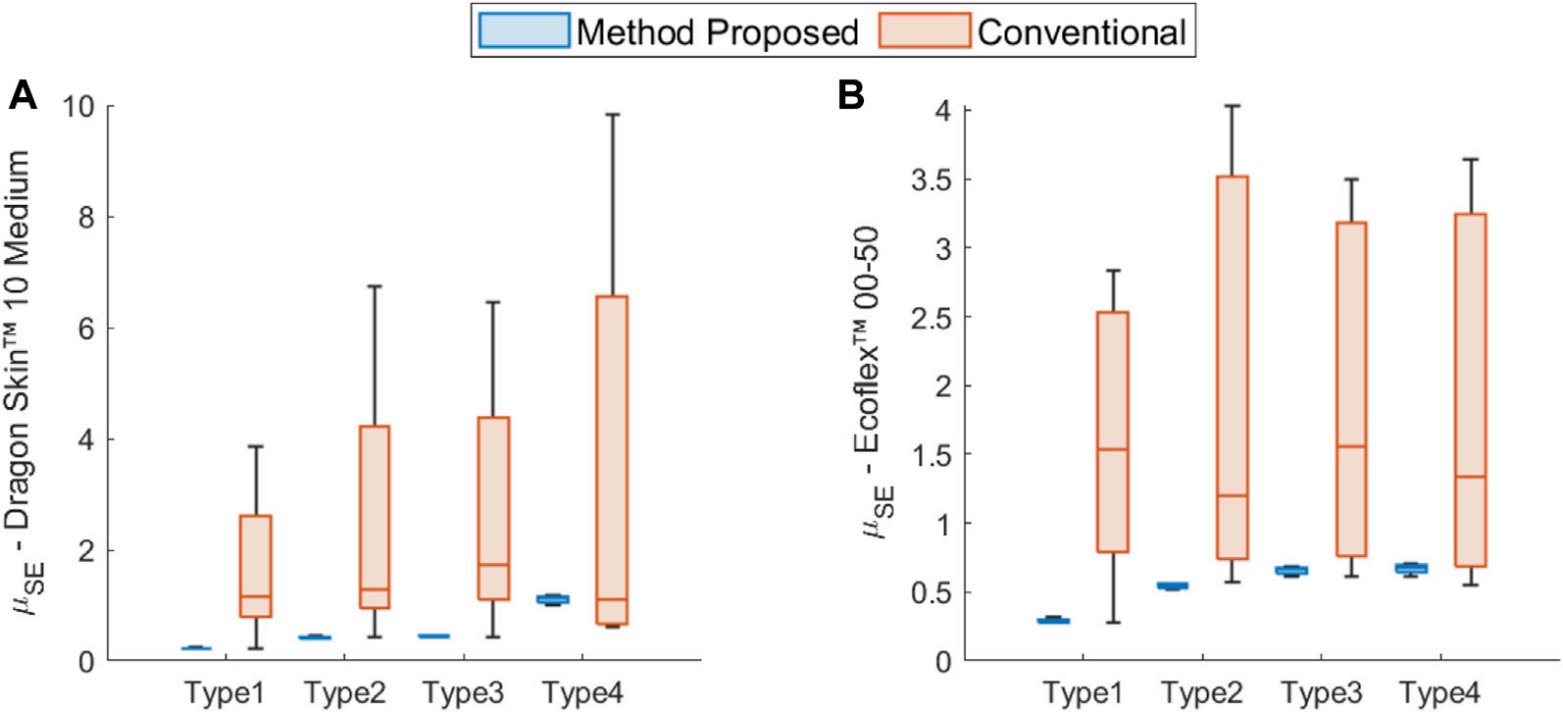
Comparison of the statistical distribution of the standard error mean (see Eq. 10) between the models obtained through the proposed EIMI and the conventional fitting approaches across the different geometry types for **(A)** Dragon Skin™ 10 Medium and **(B)** Ecoflex™ 00-50.

**FIGURE 8:**
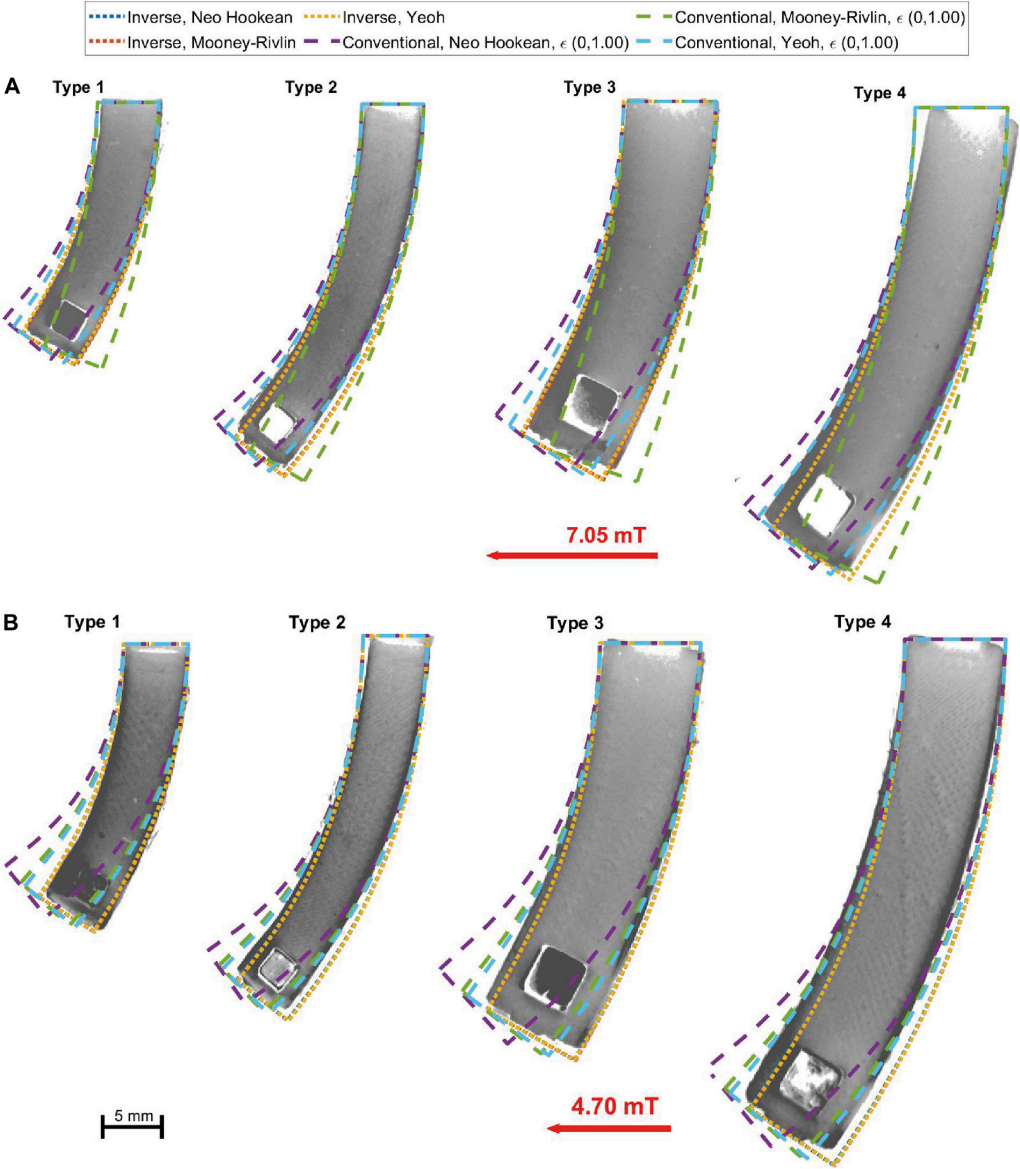
Comparison between material models and experimental images for **(A)** Dragon Skin ™ 10 Medium samples under a magnetic flux density of 7.05 mT (top half) and **(B)** Ecoflex™ 00-50 samples under a magnetic flux density of 4.7 mT (bottom half).

The results for Dragon Skin™ 10 Medium (see Table 5) show that there is a superior fit achieved for the Type 1 geometry which is the sample used for training. This result can be easily justified from the nature of the process used for characterization. The EA will tend to overfit the parameters for the sample used for training. We can also see that the standard error for the Neo Hookean, Mooney-Rivlin and Yeoh analytical models falls in the 95% confidence interval, making these analytical models similar in performance, as shown in Figure 7A. The Ogden model, however, falls outside of the confidence interval. The number of generations for the Ogden model is higher but could still be insufficient due to a higher number of parameters. To assess the generality of the model characterizations, we evaluated the errors for the other three geometries using the solution obtained by EIMI. For the Type 2 and Type 3 geometries, the standard error for the analytical models falls inside the 95% confidence interval (see Figure 7A). Finally, for the Type 4 geometry, the fitting error of the model parameters is larger. The errors may be caused by different factors, such as in segmentation of the experimental image, or an undesired torsion of the sample as observed during experimental testing (see Sample Type 4 in Figure 8A) (Lloyd et al., 2021) which is not captured within the 2D simulation.

For Ecoflex™ 00-50 (see Table 6), the proposed approach showed similar results to Dragon Skin™ 10 Medium. For the Type 1 geometry, the method showed a tendency of overfitting the models with the number of parameters less than or equal to 3. The modelling errors were also within a confidence interval of 98% (see Figure 7B). Again, the Ogden model has the worst performance for this geometry despite a fast convergence. The optimizer is able to achieve fast convergence for the Ogden model by increasing the dimension of the decision space; however, this results in a more complex landscape that is difficult to explore since more local minima can influence the search. The mean standard error ${µ}_{SE}$ falls in the 95% confidence interval for all geometries. A slight increase in the values obtained for geometries Type 2 to Type 4 (see Table 6 and Figure 7B). This result may be justified since the increase in the size of the sample points obtained by the segmentation corresponds to an enlargement of the experimental sample dimension.

### Comparison Between EIMI and the Conventional Approach

To further understand the potential of the EIMI approach, we compared it to the conventional approach of fitting material models to experimental uni-axial tensile test data. Here we take advantage of the data and the code provided by Marechal et al. (2020). For each material model analyzed (Table 1), we fit each of the hyperelastic models analyzed using the same tensile data bounded for different engineering strains. More specifically, we fit the models to consider tensile test data for strains of: 20, 50, 100% of the deformation and the full tensile dataset. The obtained parameters and associated errors are shown in the lower part of Tables 5, 6 for Dragon Skin^TM^ 10 Medium and Ecoflex^TM^ 00-50 respectively, and in Figure 7. As can be seen in both cases, the conventional approach typically produces larger errors when compared to the EIMI approach. In twenty-one of the twenty-four cases, the overall improvement with EIMI was above 26%.

For the Dragon Skin™ 10 Medium (Table 5), the Yeoh model fit *via* the conventional method with strains constrained to 50 and 100% of the original length, produced similar results to EIMI; with the latter showing modest improvement of below 2% against the best two models using the conventional approach. However, choosing the most appropriate engineering strain range for fitting purposes is not a trivial matter. The other analytical models (with different strain ranges) using the same dataset failed to get close to the same results, with the EIMI showing at least a 30% improvement. This underlines the difficulty of choosing the most suitable analytical model and the correct stress-strain selection over the tensile data.

Interestingly, for Ecoflex™ 00-50 (Table 6), the best overall performance with the conventional fitting was obtained again using the Yeoh model using the full range (16 times the original length) of strain data for Type 1 and Type 2 geometries and 100% strain for Type 3 and Type 4. Our EIMI showed a performance improvement of 6.8% compared to the best model which in this case was Ogden. For the rest of the cases, EIMI showed an improvement of at least 26%. This result again illustrates how difficult it is to pre-determine the correct range of tensile data to use in order to fit analytical models.

Overall, it is possible to see that the models fit using a conventional approach over different ranges of stress-strain data failed to converge to similar results. The box plots in Figure 7 show a higher variance between conventional models fit using the same tensile range. On the other hand, all the models obtained using the EIMI converge to similar performance values, validating the procedure. Thus, the proposed EIMI method showed that it is capable of dynamically fitting the models without any previous knowledge of the strain involved.

The performance of the different models can be clearly seen in Figure 8 where we can qualitatively observe that our proposed method shows improved performance. In both pictures, the models obtained using EIMI are compared with the models trained using tensile data with a strain of up to 100%. In general, it is possible to see that EIMI is able to produce a better result regardless of the complexity of the model, with similar responses across the different models. Whereas the model fitted using the conventional approach drifts considerably from the experimental data. For Dragon Skin^TM^ 10 (see Figure 8A), it is possible to see that the Neo Hookean and the Yeoh models underestimated the stiffness, while the Mooney-Rivlin model overestimated the stiffness. For Ecoflex^TM^ 00-50 (Figure 8B) all 3 models underestimated the stiffness.

**FIGURE 9:**
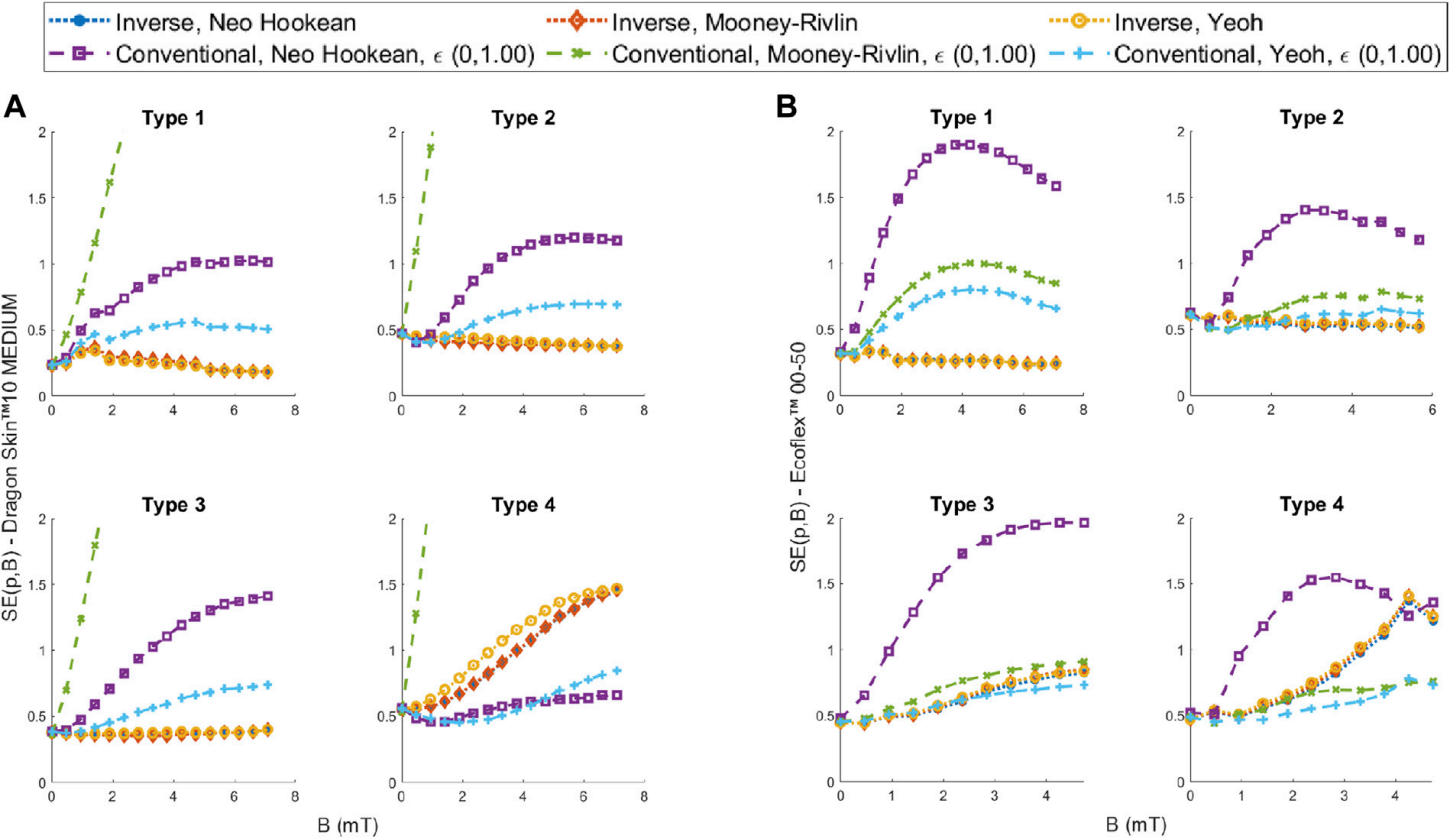
SE comparison between the models fitted using the EIMI and the conventional method using 100% of the engineering strain for increasing magnetic fields for **(A)** Dragon Skin™ 10 Medium and **(B)** Ecoflex™ 00-50.

To understand how performance varies with actuation, we analyzed EIMI and the best models found using the conventional approach for each of the magnetic field values tested. More specifically we analyzed the SE (see Eq. 9) at each magnetic field value $B_{i}$ recorded for each type of geometry, as shown in Figure 9. This allowed us to understand how the models drift from the experimental sample with increasing magnetic torque.

The performance of the Dragon Skin™ 10 Medium is shown in Figure 9A. For geometry Types 1, 2 and 3, all the models obtained using our EIMI approach produce a smaller error than the best performer (Yeoh) from the conventional approach. Furthermore, the error is constant with the increasing magnetic field, while for the other models it increases with the magnetic field. For Type 4, we see that the conventional Yeoh and Neo Hookean models have a smaller error across all field values. This was due to an observed twisting in the sample that reduced the bending of the beam, which can be interpreted as a stiffer material in the 2D plane (see Supplementary Video S1). This is why we see the errors obtained using the EIMI approach steadily increasing for the Type 4 geometry.

For Ecoflex™ 00-50 (see Figure 9B), we can see that for the sample used for training (Type 1) the models obtained using EIMI outperform all other models. For Types 1 and 2 samples, the Yeoh and Mooney-Rivlin models fitted over 100% strain of tensile data showed slightly worse performance compared to the EIMI and have a similar trend in the SE with the increasing magnetic field. However, these models outperform the EIMI for higher values of the magnetic field in Type 4, again likely due to the twisting of the sample observed experimentally (see Supplementary Video S1).

Overall, our proposed approach is able to maintain a steady error (Figure 9) with increasing magnetic field showing that it is able to dynamically follow the deflection of the robot.

## Conclusion and Future Development

In this work, we presented an alternative method of material characterization to dynamically find the best material model based on the target application. We present a framework for characterizing hyperelastic materials using an FEA simulation, application-specific experimental evaluation and an EA to solve the optimization problem. The results show that the proposed EIMI approach can fit the parameters for a material model with a high degree of accuracy. Furthermore, thanks to the use of the simulation model in the characterization process, we can intrinsically obtain robust and stable models to use with numerical methods.

Our results also highlight the inherent challenges with the conventional model-fitting approach. In this study, we rely on the database of Marechal et al. (2020), where the models are fitted by minimizing the standard error between the experimental data and simulation to allow the reader to easily verify and evaluate new models. However, other strategies such as the minimization of the relative errors may be considered to improve the material characterization (Destrade et al., 2017) or the use of a genetic algorithm (López-Campos et al., 2019). In addition, while it is possible to get good results with a correct model and strain range selection for a given application, knowing these in advance is not always possible, and incorrect selections can lead to large errors. In contrast, the proposed EIMI approach allows us to dynamically find the best fit, without limiting and testing the models recursively and without knowing the scale of the strain involved.

Despite this success, some limitations to our approach exist. When it is possible equiaxial and volumetric testing are generally preferred (Mihai and Goriely, 2017). Effects of viscoelasticity and compressibility were not considered in this study. Compressibility can be easily integrated into the framework presented here by enlarging the search space. On the other hand, viscoelasticity may require careful experimental design, since it is a transient property. Between each step of induced stress, the time necessary to reach the equilibrium will need to be precisely recorded. It will also be necessary to replicate the experiment using a time-dependent FEM simulation. Lastly, only one model that uses the second invariant $I_{2}$ of the Cauchy Tensor has been used (Mooney-Rivlin). In the future it might be beneficial to include others like the Gent-Gent model (Pucci and Saccomandi, 2002; Anssari-Benam et al., 2021).

While EIMI allows the user the freedom to choose any actuation method, we focused on magnetic actuation in this study which is often approximated using the dipole model, and this may not always be accurate. An oversimplification of the physical model for the simulation will negatively impact the characterization and drift the model parameters from their true values. However, if the experimental setup is similar to the final application the error caused by the discretization should be contained.

While in this study we focus only on magnetic actuation, the EIMI approach may be expanded and used to validate other actuation schemes such as pneumatic or piezoelectric, with suitable testbed and simulation development. In addition, the decision space may be further expanded by adding more parameters to the characterization such as the magnitude of the actuation forces. For example, if the magnetic moment is unknown, we can include this variable in the decision space with the soft material parameters. In this case, the EA would need to solve an extra degree of freedom to match the experimental data. Finally, while the analytical model is usually the result of empirical observation, we can try to expand the optimization problem by also including the strain energy function. With enough computational power, it may be possible to use a technique such as genetic programming (Affenzeller et al., 2009) to determine the best ad-hoc analytical equation that expresses the strain energy function in parallel to the parameter fitting operation.

## Data Availability

The raw data supporting the conclusions of this article will be made available by the authors, without undue reservation.

## Supplementary Material

Supplementary Video S1 Visual comparison between the models fitted using EIMI and conventional methods, using 100% of the engineering strain for increasing magnetic fields with respect to the experimental images obtained with the Helmholtz coil for Dragon Skin™ 10 Medium and Ecoflex™ 00-50.
